# Eating in the Cars

**Published:** 1871-07

**Authors:** 


### EATING IN THE CARS.

Most of the benefits of summer travel and recreation are over-balanced by the almost universal habit of passengers in railway trains of purchasing something to eat of nearly every peddler of lozenges, candies, apples, cakes, and other trash, who passes through the cars, with the result of leaving but a little appetite for the regular meal, besides a general indefinable feeling of discomfort, of wanting something, they know not what.

Parents of small children seem to think that the best way to keep them from eternal yelping is to stuff them with sweet cakes and candies, and as fast as one supply is disposed of another is provided, making such a mess on the floor and seats as would disgrace a common pig-pen or hen-coop. By providing sweet cakes and candies, thirst is induced, then fullness, then indigestion, wind, and a universal caterwauling of squalling brats, who ought to be spanked within an inch of their lives; a single yauper being enough to keep a car load of sixty or a hundred travellers in a disturbed condition. Young children on the cars should not be allowed to eat anything but dry crackers; then they would not grease the seats, nor eat to excess, nor be squawking with the stomach-ache half the time; and as for grown persons, not an atom should be eaten all day long, except at morning, noon, and night meals.

